# The Native Copper- and Zinc- Binding Protein Metallothionein Blocks Copper-Mediated Aβ Aggregation and Toxicity in Rat Cortical Neurons

**DOI:** 10.1371/journal.pone.0012030

**Published:** 2010-08-11

**Authors:** Roger S. Chung, Claire Howells, Emma D. Eaton, Lana Shabala, Kairit Zovo, Peep Palumaa, Rannar Sillard, Adele Woodhouse, William R. Bennett, Shannon Ray, James C. Vickers, Adrian K. West

**Affiliations:** 1 NeuroRepair Group, Menzies Research Institute, University of Tasmania, Hobart, Australia; 2 Department of Gene Technology, Tallinn Technical University, Tallinn, Estonia; Mental Health Research Institute of Victoria, Australia

## Abstract

**Background:**

A major pathological hallmark of AD is the deposition of insoluble extracellular β-amyloid (Aβ) plaques. There are compelling data suggesting that Aβ aggregation is catalysed by reaction with the metals zinc and copper.

**Methodology/Principal Findings:**

We now report that the major human-expressed metallothionein (MT) subtype, MT-2A, is capable of preventing the *in vitro* copper-mediated aggregation of Aβ_1–40_ and Aβ_1–42_. This action of MT-2A appears to involve a metal-swap between Zn_7_MT-2A and Cu(II)-Aβ, since neither Cu_10_MT-2A or carboxymethylated MT-2A blocked Cu(II)-Aβ aggregation. Furthermore, Zn_7_MT-2A blocked Cu(II)-Aβ induced changes in ionic homeostasis and subsequent neurotoxicity of cultured cortical neurons.

**Conclusions/Significance:**

These results indicate that MTs of the type represented by MT-2A are capable of protecting against Aβ aggregation and toxicity. Given the recent interest in metal-chelation therapies for AD that remove metal from Aβ leaving a metal-free Aβ that can readily bind metals again, we believe that MT-2A might represent a different therapeutic approach as the metal exchange between MT and Aβ leaves the Aβ in a Zn-bound, relatively inert form.

## Introduction

Alzheimer's disease (AD) is the most common form of dementia within the ageing population. AD accounts for between 50% and 60% of dementia cases [1]. The pathological hallmarks of the disease include extracellular β-amyloid (Aβ) plaques, intracellular neurofibrillary tangles and dystrophic neurites [2]. All of these hallmarks arise from the abnormal and unregulated overproduction of insoluble proteinaceous structures. These proteinaceous structures are responsible for the disruption to normal cellular functioning ultimately leading to cell death.

The main constituents of Aβ plaques are the 40- and 42-mer peptides, Aβ_1–40_ and Aβ_1–42_ [3], which associate to form abnormal extracellular deposits of fibrils and amorphous aggregates [4]. Aβ is derived from the β-amyloid precursor peptide (APP), which is a normal protein [5], [6] that is produced by neuronal and non-neuronal cells [7], [8]. APP is processed by a combination of α-, β-, and/or γ- secretases to form numerous protein products. The plaque-forming Aβ_1–42_ and Aβ_1–40_ arise from the uncommon β-and γ-secretase cleavage of the APP.

The mechanisms underlying the aggregation of Aβ have been the subject of intense investigation. There is compelling data to suggest that the aggregation of Aβ is catalysed by reaction with the metals zinc and copper. Cu-mediated aggregation of Aβ leads to the formation of copper-bound, SDS-insoluble Aβ aggregates, that are neurotoxic due to the ability of Aβ-bound copper to undergo redox reactions at the cell membrane to generate reactive oxygen species [9], [10]. Of note, Aβ plaques deposited in the AD brain are found to be enriched with metals, and in particular copper [11], [12].

Metallothioneins (MTs) are the major endogenous zinc- and copper- binding protein within the brain. The MT-1/2 isoforms (exemplified by the highly expressed member, MT-2A) are characterised as highly neuroprotective proteins essential for brain repair [13]–[15]. The metal-binding properties of MT-1/2 have been well investigated, and it is recognised that these proteins are capable of binding 7 divalent (Zn^2+^) and up to 12 monovalent (Cu^+^) metal ions *in vivo* through two distinct metal-thiolate clusters, termed the α- and β- domains [16]. The importance of MT in maintaining metal homeostasis is clearly demonstrated in studies involving exposure to heavy metals (either by diet or environment) in MT-1/2 knockout mice, which leads to metal toxicity, while MT-1/2 overexpressing mice are relatively protected from heavy metal toxicity [see review, 17]. MTs also have important roles in copper homeostasis, evidenced by the crossing of a mouse model of Menkes disease (a copper efflux disease) with MT-1/2 knockout mice, which results in embryonic lethality [18].

Given the strong metal-binding properties of MT, we predict that these proteins may be involved in regulating the metal-binding and subsequent aggregation of Aβ. Indeed, there is substantial literature supporting a role for MTs in the pathophysiology of AD. These proteins are expressed by astrocytes, and their expression is significantly upregulated in regions of Aβ plaque pathology in the pre-clinical [19] and clinical AD brain [20], [21], as well as in the brains of transgenic AD mice [22]. Notably, Meloni et al [23] recently reported that the Zn(II)-MT-3 isoform is capable of exchanging metals with Cu(II)-Aβ to prevent the formation of SDS-insoluble Cu(II)-Aβ aggregates. The goal of this study is to evaluate whether MT-2A, via its zinc- and copper- binding properties, also represents an endogenous protective mechanism against Aβ aggregation and toxicity.

## Results

### MT-2A prevents copper-mediated formation of SDS-insoluble Aβ aggregates

**Figure 1:**
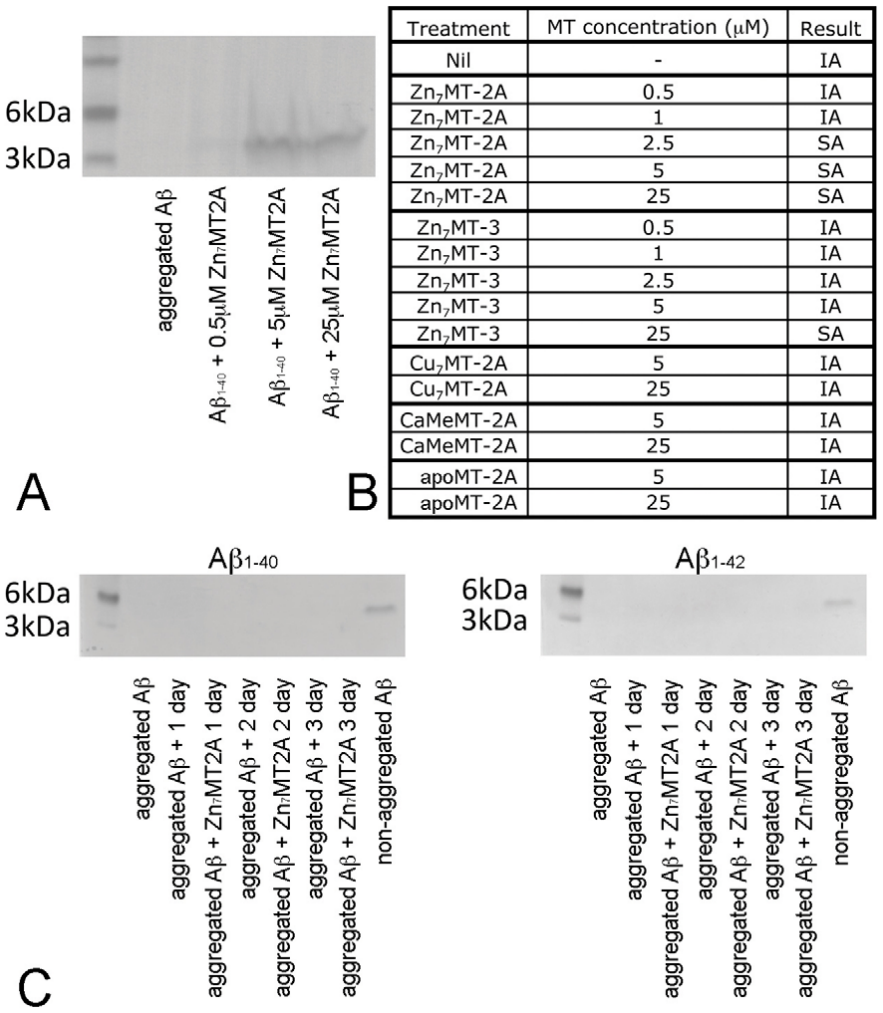
25µM Aβ_1–40_, in the presence of 25µM copper and 200µM ascorbate, was incubated at 37°C for three days with shaking at 300rpm. This resulted in the formation of SDS-insoluble Aβ aggregates (IA), which were not visualised on SDS-PAGE (A). The presence of Zn_7_-MT2A (5–25µM) prevented formation of SDS-insoluble Aβ aggregates (A). Instead, SDS-soluble aggregates (SA) were formed, that were resolved as a single protein band of approximately 3–4kDa size, representing monomeric Aβ_1–40_ peptide (A). A range of different MT forms were tested for the ability to promote formation of SDS-soluble aggregates (SA)(B). Zn_7_MT-2A promoted formation of SDS-SA, and Zn_7_MT-3 had a similar effect but at 10-fold higher concentration (B). The ability of MT-2A to prevent formation of SDS-IA was linked to metal-binding properties, as different metallated forms of MT-2A had different effects upon Aβ aggregation (B). When Cu-Aβ_1–40_ or Cu-Aβ_1–42_ aggregates were generated by three days of incubation, and then incubated with shaking in the presence or absence of 25µM Zn_7_MT-2A for up to three days, this was unable de-aggregate either Cu-Aβ_1–40_ or Cu-Aβ_1–42_ pre-formed aggregates (C).

To stimulate Aβ aggregation, 25µM of Aβ_1–40_ was mixed with an equimolar concentration of copper and 200µM ascorbate, and shaken at 300rpm for three days at 37°C. The resultant protein aggregates were collected by ultracentrifugation, resuspended in SDS-PAGE loading buffer and electrophoresed. Under these conditions, no Aβ_1–40_ was visualised on SDS-PAGE (Figure 1A), as a consequence of the formation of copper-bound SDS-insoluble aggregates (IA). In contrast, in the presence of Zn_7_MT-2A at the range of 2.5–25µM, Aβ_1–40_ formed aggregates (SA)(Figure 1B), which dissolved in SDS and resolved as a single band on SDS-PAGE of approximately 3–4kDa representing monomeric Aβ_1–40_ (Figure 1A). As shown recently by Meloni et al [23] the structurally related metallothionein isoform Zn_7_MT-3 was also capable of preventing formation of SDS-insoluble Aβ_1–40_ aggregates, but required a 10-fold higher concentration than Zn_7_MT-2A (Figure 1B). We also investigated whether Zn_7_MT-2A can prevent the Cu-mediated aggregation of Aβ_1–42_. Under the same experimental conditions, 25µM Zn_7_MT2A was able to completely prevent Cu-Aβ_1–42_ forming SDS-insoluble aggregates (results not shown).

We also investigated whether MT-2A can de-aggregate pre-formed Cu-Aβ aggregates. Cu-Aβ_1–40_ and Cu-Aβ_1–42_ aggregates were produced as described above (three day incubation), after which time 25µM of Zn_7_MT-2A was added. However, incubation with Zn_7_MT-2A for up to three days was unable to de-aggregate either Cu-Aβ_1–40_ or Cu-Aβ_1–42_ pre-formed aggregates (Figure 1C).

To establish whether the metallation state of MT is responsible for the ability of MT-2A to prevent aggregation of Aβ_1–40_ into SDS-IA, several different metallated forms of MT-2A were used. We found that Cu_10_MT-2A was not able to prevent the formation of SDS-insoluble Aβ_1–40_ aggregates (Figure 1). Furthermore, chemical modification of MT-2A to block metal binding, by means of carboxymethylation of cysteine residues (CaMeMT-2A) abolished the ability of MT-2A to prevent formation of insoluble Aβ_1–40_ aggregates (Figure 1). Finally, metal free (apo) MT-2A was also unable to prevent copper-mediated formation of insoluble Aβ_1–40_. Based upon these observations, we predict that the zinc bound to MT is required for the ability of MT to block copper-mediated Aβ_1–40_ aggregation.

### Evidence that Zn_7_MT-2A prevents copper-mediated Aβ aggregation via metal exchange

One possible explanation for our observations is that there is a metal exchange of copper and zinc between Zn_7_MT-2A and Cu(II)Aβ with the subsequent formation of Zn-bound Aβ aggregates and soluble Cu-bound MT. To test this hypothesis, the amount of copper and zinc present in the aggregated Aβ samples was determined using inductively coupled plasma mass spectrometry (ICP-MS). When Aβ_1–40_ was aggregated for three days in the presence of copper and ascorbate, the amount of copper/zinc detected within the pellet fraction following ultracentrifugation was 89.2/27.1ng (Table 1). However, when Aβ_1–40_ was aggregated in the presence of Zn_7_MT-2A, the amount of copper present in the pellet fraction was significantly reduced to 47.5ng (approximately 53% decrease), while there was a concomitant increase in zinc to 81.4ng (Table 1). There was no change in copper/zinc levels however when CaMeMT-2A was used (Table 1). This provides further evidence that a copper-zinc exchange has occurred between Cu(II)-Aβ and Zn_7_MT-2A. We therefore predict that the action of MT-2A to prevent the formation of insoluble Aβ aggregates is due to metal exchange of copper and zinc between Zn_7_MT-2A and Cu(II)Aβ with the subsequent formation of Zn-bound Aβ which only forms soluble protein aggregates.

**Table 1:** 25µM Aβ_1–40_ was mixed with 25µM copper and 200µM ascorbate (rapidly forming Cu(II)-Aβ), and incubated with shaking at 37°C for 72 hours, resulting in the formation of aggregated Aβ_1–40_.

Reaction	Copper (ng)	Zinc (ng)
40mM Cu(II)-Aβ1–40	89.2	27.1
40mM Cu(II)-Aβ1–40+25mM Zn7MT-2A	47.5*	81.4*
40mM Cu(II)-Aβ1–40+25mM CaMeMT-2A	87.9	27

Pellets containing Aβ_1–40_ were collected by ultracentrifugation, and the content of copper and zinc in the pellet samples was measured by ICP-MS. All samples were performed in triplicate, and the average presented. Cu(II)-Aβ_1–40_ aggregation in the presence of Zn_7_MT-2A resulted in a significant reduction in the amount of copper (and increase in zinc) present in the aggregated Aβ_1–40_ pellet in comparison to Cu(II)-Aβ_1–40_ aggregated in the absence of MT-2A (p<0.05, t-test).

### Comparative measurement of the relative Cu(I)-binding affinity of MT-2A with MT-3

**Figure 2:**
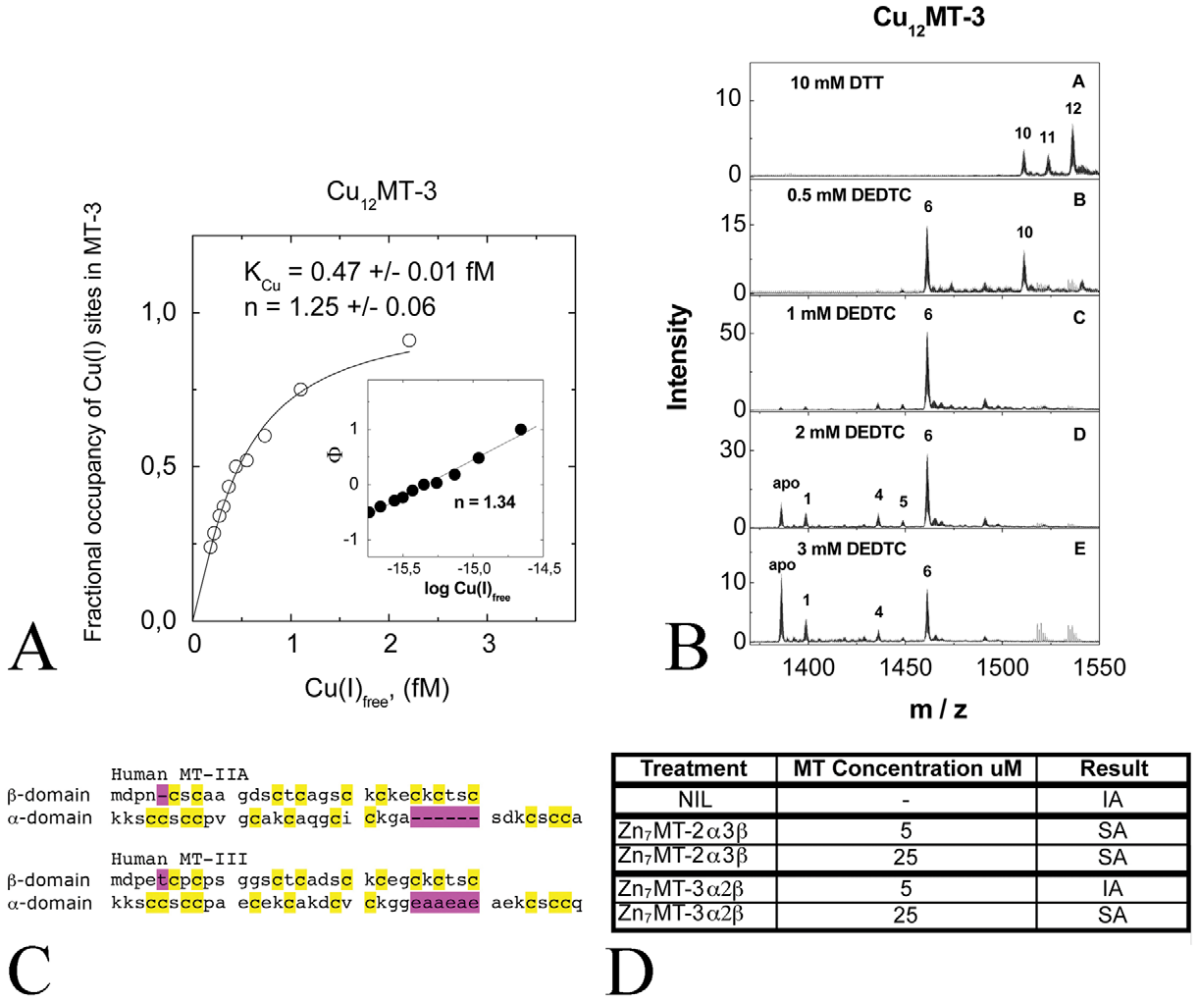
Using ESI-MS, we determined that the Cu(I)-binding affinity of MT-3 (K_Cu_) is equal to 0.47±0.01 fM)(A). To investigate the binding affinities of the individual copper-thiolate clusters of MT-3, we exposed Cu_12_MT-3 to the high affinity Cu(I)-binding chelator DETC (B). At 0.5 mM concentration DETC, Cu_12_MT-3 converted to a predominant Cu_6_MT-3 form, which could be further demetallated to apo-MT-3 at 3 mM DETC (B). Human MT-3 shares approximately 60% amino acid sequence homology with human MT-2A (C). Conserved cysteines are highlighted in yellow, and amino acid insertions indicated in purple. Zn_7_MT-3β2α was able to prevent formation of SDS-insoluble Aβ_1–40_ aggregates (IA), while Zn_7_MT-2β3α could only prevent insoluble aggregate formation at a concentration of 25µM (D).

An explanation for the different abilities of MT-2A and MT-3 to alter Cu(II)-Aβ aggregation might lie in different copper-binding properties of MT-2A and MT-3. Formal thermodynamic analysis indicated that MT-3 has only slightly lower apparent Cu(I)-binding affinity (K_Cu_ = 0.47±0.01 fM) (Figure 2A) as compared with that which we have recently reported for MT-2A (K_Cu_ = 0.41±0.02 fM) [24]. However, differences were observed in the composition and affinities of individual copper-thiolate clusters of MT-2 and MT-3. In ESI-MS studies in the presence of 10mM DTT, we observed that copper was bound to MT-3 in a mixture of major Cu_12_MT-3 and minor Cu_10_MT-3 forms (Figure 2B), while we have recently reported that MT-2A is predominantly found in Cu_10_MT-2A form [24]. Addition of the high affinity Cu(I)-binding chelator DETC at 0.5 mM concentration was sufficient to convert Cu_12_MT-3 to a predominant Cu_6_MT-3 form, which could be further demetallated to apo-MT-3 at 3 mM DETC (Figure 2B). These observations indicate that MT-3 binds copper in two distinct hexacopper clusters exposing different Cu(I)-binding affinities. Conversely however, Cu_10_MT-2A was stable at up to 1.0 mM DETC, and that 1.5mM DETC was required to partially demetallate Cu_10_MT-2A to Cu_6_MT-2A [24]. This suggests that tetracopper-thiolate cluster in Cu_10_MT-2A has higher Cu(I)-binding affinity than low-affinity hexacopper-thiolate cluster in Cu_12_MT-3, and may account for the greater ability of MT-2A to prevent Cu(II)-Aβ aggregation compared to MT-3. We predict from the amino acid composition of MT-2A and MT-3 that due to their high sequence homology that the N-terminal β-domains will share similar metal-binding properties (Figure 2C), most likely corresponding to the hexacopper-thiolate cluster that is demetallated by treatment with 3mM DETC. Subsequently, the C-terminal α-domain is likely to represent the metal thiolate cluster that differs between the two isoforms and which may contribute to the different copper-binding properties of MT-2A and MT-3. To test this hypothesis we switched the domains between isoforms to form chimeric recombinant MT-3β2α and MT-2β3α proteins (note the the beta-domains of each chimera are at the N-terminus, which is the same as native MTs). Indeed, Zn_7_MT-3β2α was capable of preventing copper mediated Aβ aggregation to a similar degree as Zn_7_MT-2A, while the Zn_7_MT-2β3α chimeric protein had a similar activity to Zn_7_MT-3 (Figure 2D).

### MT-2A protects against Aβ toxicity in cultured cortical neurons

**Figure 3:**
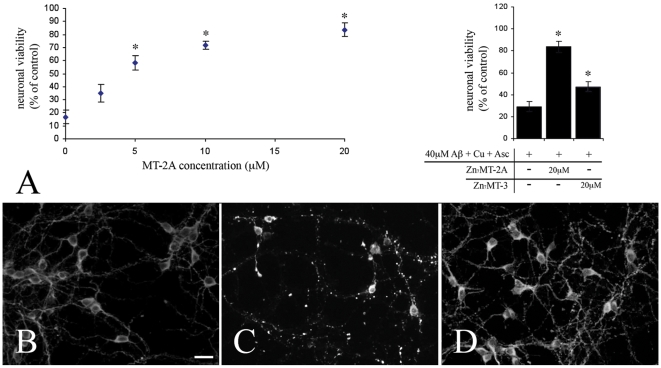
Treatment of 3DIV rat cortical neuron cultures with 40µM Aβ_1–40_ (in the presence of 40µM CuCl_2_ and 300µM ascorbate) resulted in approximately 80% reduction in neuronal viability 24 hours after treatment (A). 20µM of Zn_7_MT-2A was far more effective in protecting against Aβ_1–40_ neurotoxicity than 20µM Zn_7_MT-3 (A). Immunolabelling for tau demonstrated smooth cytoskeletal labelling in control (B) neurons, which became punctate and disassociated following Cu(II)Aβ_1–40_ treatment (C). Treatment with 20µM Zn_7_MT-2A prevented Aβ-induced structural changes in the neuronal cytoskeleton (D). Scale bar = 25mm.

It has been reported previously that Cu(II)-Aβ_1–40_ but not Zn(II)-Aβ_1–40_ is toxic to cultured neurons [12], [25]. The combination of equi-molar concentrations of Aβ_1–40_ and Cu(II) ions results in all free copper becoming rapidly bound to Aβ_1–40_ to form Cu(II)-Aβ_1–40_ [23]. We maintained rat cortical neurons for three days *in vitro* (3DIV) followed by treatment with 40µM Cu(II)-Aβ_1–40_ and 300µM ascorbate. After 24 hours, this treatment resulted in about an 80% reduction in cell viability, as measured using an alamarBlue® cell viability assay (Figure 3A). In parallel experiments we performed direct cell counts to validate the alamarBlue® data, and observed a comparable degree of neurotoxicity in the presence of 40mM Cu(II)-Aβ_1–40_ (Figure S1). We found that Zn_7_MT-2A blocked Aβ-induced toxicity in a dose dependent manner (5–20µM range), with almost complete protection at a concentration of 20µM Zn_7_MT-2A (Figure 3A). Furthermore, 20µM of Zn_7_MT-2A was far more effective in protecting against Cu(II)-Aβ neurotoxicity than 20µM Zn_7_MT-3 (Figure 3A). Immunolabelling of neurons for the cytoskeletal protein tau demonstrated that Zn_7_MT-2A protected against Cu(II)-Aβ induced neuronal degeneration (Figure 3B–D). Treatment with ascorbate or Aβ_1–40_ alone had no effect upon viability (results not shown). Note that addition of MT-3 to neuronal cultures can have powerful neurotoxic effects in its own right, if applied in the presence of a brain derived extract or serum [26], [27], neither of which are present here. In contrast, in the absence of these agents, MT-3 alone has no discernible neurotoxic effect.

**Figure 4:**
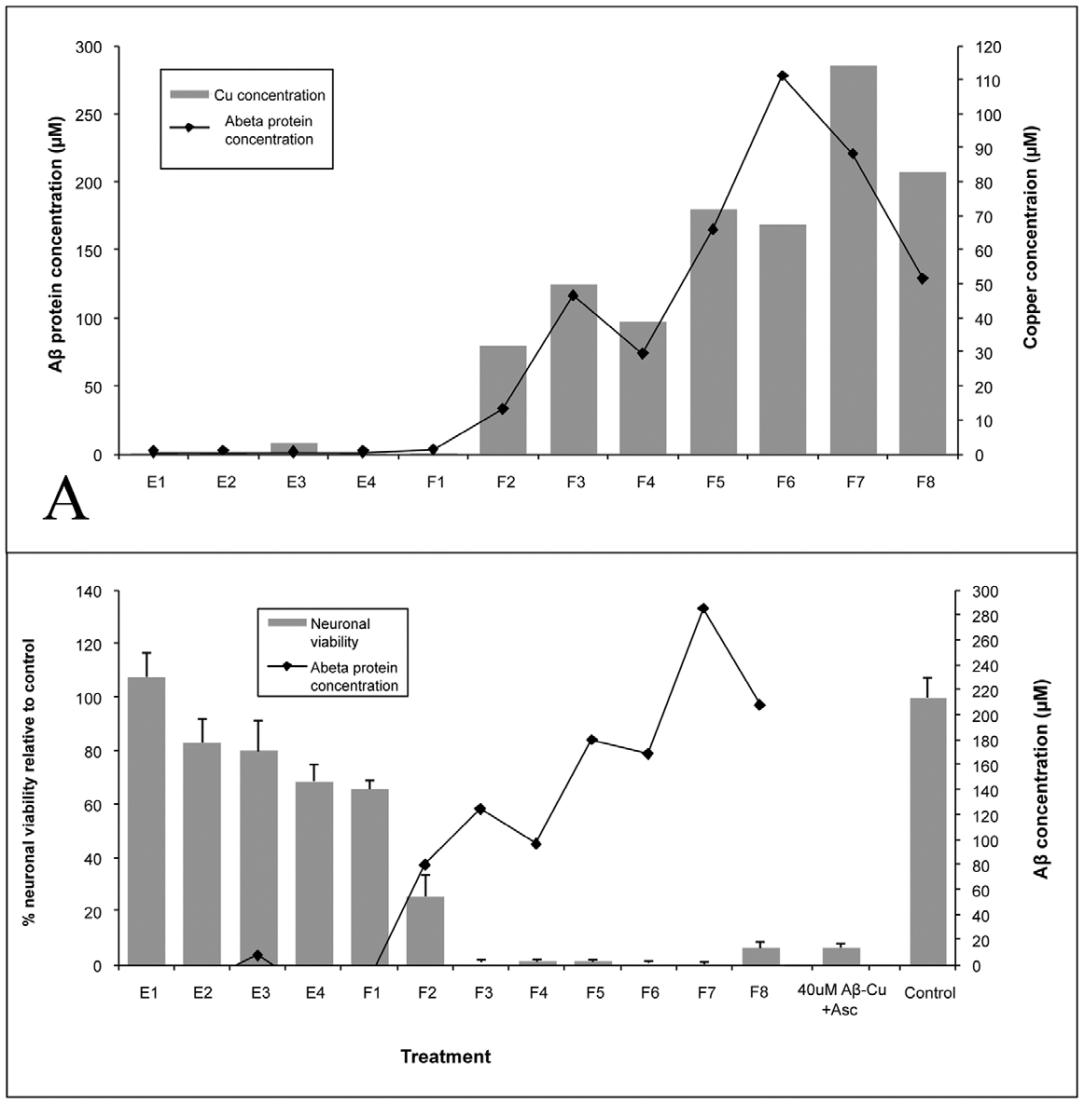
A 40µM Cu(II)-Aβ_1–40_ solution was separated on a PD MidiTrap™ G-25 column, and the protein (A280) and metal (ICP-MS) content of each fraction determined (A). Only those fractions containing Aβ peptide also contained copper (A), indicating that the Cu(II)-Aβ_1–40_ solution did not contain any free copper ions. The neurotoxicity of all fractions was subsequently tested, and only those containing Cu(II)-Aβ_1–40_ exhibited neurotoxic activity (B). E – eluate, F – collected fraction.

To further investigate whether it is Cu(II)-Aβ and not free copper that is responsible for neurotoxicity, we fractionated a 40µM Cu(II)-Aβ solution on a PD MidiTrap™ G-25 column and determined the protein (A280) and metal (ICP-MS) content of each fraction. Only those fractions containing peptide also contained copper (Figure 4A), indicating that the Cu(II)-Aβ solution did not contain any free copper ions. The neurotoxicity of all fractions was subsequently tested, and only those containing Cu(II)-Aβ exhibited neurotoxic activity (Figure 4B), demonstrating that Cu(II)-Aβ is responsible for the neurotoxicity that we have observed in our experiments.

**Figure 5:**
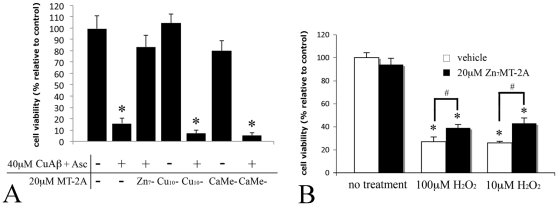
Treatment with 20µM Cu_10_MT-2A or CaMeMT-2A had no protective effect against 40µM Aβ_1–40_ (in the presence of 40µM CuCl_2_ and 300µM ascorbate) (A). Treatment of cortical neurons with 10µM or 100µM H_2_O_2_ for two hours resulted in substantial neuronal death, which was not prevented by co-treatment with 20µM Zn_7_MT-2A (B). Error bars represent standard error of the mean calculated from at least three experimental replicates. In panel A, * - p<0.05 compared to untreated cells (One-Way ANOVA). In panel B, Two-Way ANOVA analysis was performed, and * - p<0.05 compared to untreated cells, # - p<0.05 between treatments.

To investigate whether MT-2A acts protectively via the zinc-copper metal exchange between Zn_7_MT-2A and Cu(II)-Aβ described above, Cu_10_MT-2A was used in the place of Zn_7_MT-2A, which resulted in no neuroprotection against Cu(II)-Aβ (Figure 5A). This suggests that the metal exchange between MT-2A and Cu(II)-Aβ is required for neuroprotection. Notably, Cu_10_MT-2A alone was not toxic to neurons (Figure 5A), confirming that when copper is bound to MT it is unable to produce ROS. In parallel experiments, we found that Zn_7_MT-2A was only able to mildly block the neurotoxicity of H_2_O_2_ when applied to cultured neurons (Figure 5B). The amount of H_2_O_2_ directly applied to the neurons was physiologically relevant as Huang and colleagues [28] found that 10µM Aβ_1–40_ or Aβ_1–42_ may generate up to 25µM H_2_O_2_ in 1 hour in the presence of substoichiometric amounts of Cu(II), depending on the oxygen tension. Since H_2_O_2_ is the direct ROS product of the interaction of Cu(II)-Aβ with cells [23], this suggests that the protective action of Zn_7_MT-2A is primarily upstream of ROS production. This is consistent with our results suggesting that Zn_7_MT-2A acts via a metal swap with Cu(II)-Aβ to prevent ROS formation.

**Figure 6:**
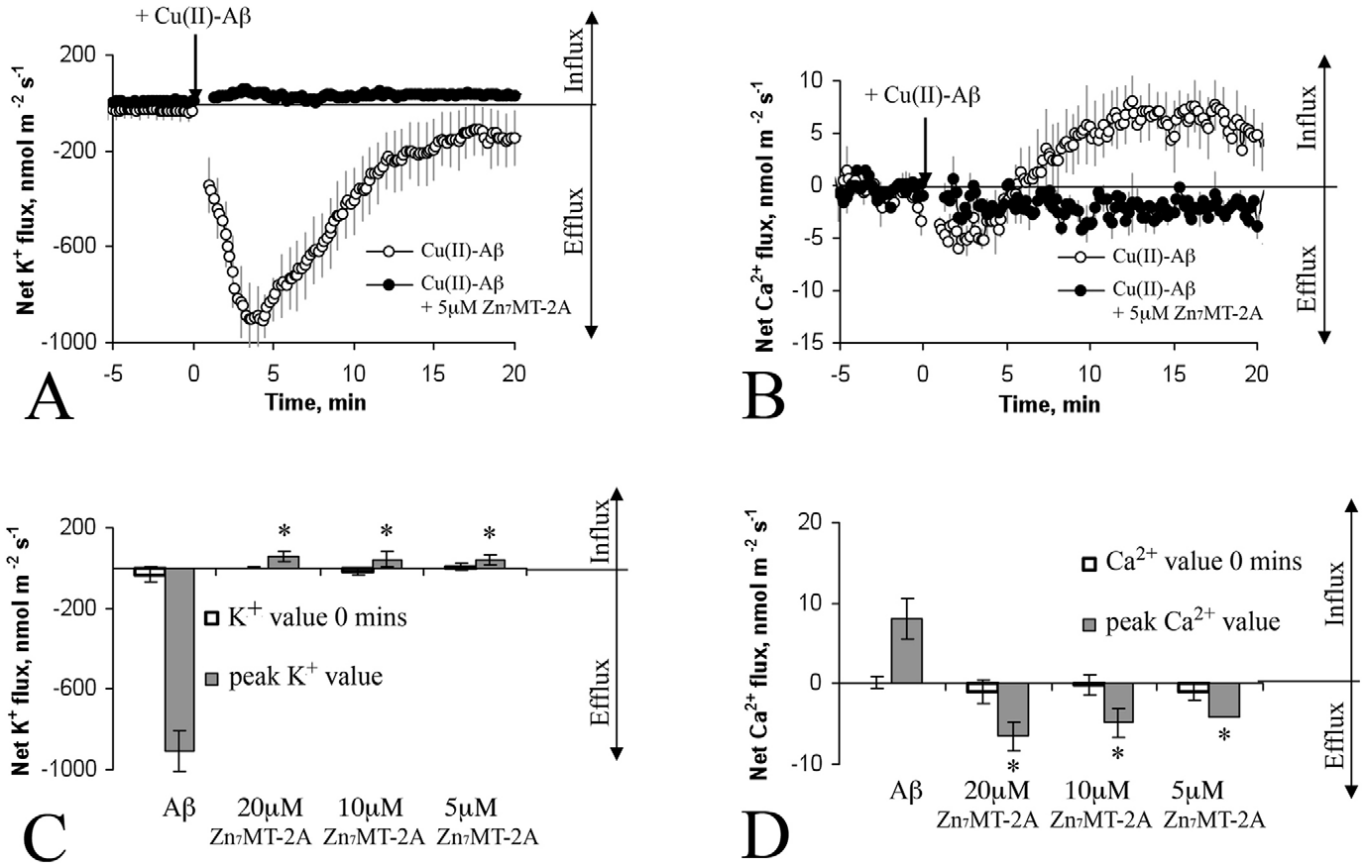
A non-invasive microelectrode ionic flux measuring (MIFE) technique was used to measure the kinetics of K^+^ and Ca^2+^ fluxes simultaneously in response to Cu(II)-Aβ and Zn_7_MT-2A. Treatment with 40µM Cu(II)Aβ_1–40_ resulted in a massive efflux of K^+^ out of neurons (A), and Ca^2+^ influx into neurons (B). Quantification of net ion flux revealed that co-treatment with Zn_7_MT-2A completely blocked Cu(II)Aβ-induced changes in K^+^ (A and C) and Ca^2+^ fluxes (B and D), at a concentration of >5mM. For all MIFE data, the sign convention is “influx positive”. Error bars represent standard error of the mean calculated from at least three different experiments. * - p<0.05 compared to Cu(II)-Aβ treated cells (One-Way ANOVA).

To further confirm that Zn_7_MT-2A is blocking the detrimental effects of oxidative stress induced by Cu(II)-Aβ, we have measured changes in ionic homeostasis of neurons in response to Cu(II)-Aβ using a microelectrode ion flux measuring (MIFE) technique. The MIFE technique allows direct measurement in changes in K^+^ and Ca^2+^ fluxes in response to Cu(II)-Aβ. Treatment with Cu(II)Aβ_1–40_ (in the presence of ascorbate) induced a rapid efflux of K^+^ out of neurons, peaking at 4.2±0.65 min after Cu(II)-Aβ application with K^+^ outflow continuing over a period of 20 minutes (Figure 6A). The treatment also led to a moderate influx of Ca^2+^, peaking at 6.45±1.35 min after the Cu(II)-Aβ application (indicated by crossing zero line) with Ca^2+^ uptake continuing over the experimental period (Figure 6C). Treatment with Zn_7_MT-2A completely blocked Cu(II)-Aβ induced changes in K^+^ and Ca^2+^ fluxes, at a concentration of >5µM (Figure 6B, D). Individual treatments with either Aβ_1–40_ or Zn_7_MT-2A alone (in the presence of ascorbate) had no effect upon any of the ions measured (results not shown).

## Discussion

The major findings of this study are that the major human-expressed subtype of metallothionein, MT-2A, is capable of preventing the formation of the toxic Cu-mediated aggregates of Aβ_1–40_ and Aβ_1–42_. This action of MT-2A appears to involve a metal-swap between Zn_7_MT-2A and Cu(II)-Aβ since neither Cu_10_MT-2A or carboxymethylated MT-2A blocked Cu(II)-Aβ aggregation. Furthermore, Zn_7_MT-2A blocked Cu(II)-Aβ_1–40_ induced neurotoxicity of cultured cortical neurons. We propose that there is therapeutic potential in a MT-2A based approach to reducing Aβ deposition in AD.

It is well established that MT-1/2 expression is elevated in response to almost all forms of stress to the brain, including traumatic-, ischaemic or chemical- brain injury [25], [29], [30], or in neurodegenerative conditions such as ALS or EAE (an experimental animal model of multiple sclerosis). The common consensus is that MT-1/2 act neuroprotectively, via intracellular functions such as metal detoxification and quenching of oxidative free radicals. More recently, it has been demonstrated that MT-1/2 can be actively secreted by astrocytes under certain pathophysiological conditions [15], and subsequently act from an extracellular location directly upon neurons to activate intracellular neuroprotective pathways [15], [31], [32]. However, an unexpected and specific protective function of MT in the AD brain has recently been proposed by Meloni and colleagues [23], who have found that Zn_7_MT-3 is able to prevent copper-mediated aggregation of Aβ *in vitro*, and protect a neuronal cell line from soluble Aβ_1–40_ toxicity. There is some controversy over the level of expression of MT-3 in the AD brain, although it appears that expression is downregulated in AD. Furthermore, the expression of MT-3 is not induced by metals or oxidative stress, and this protein displays neurotoxic actions under some conditions [14], [26], suggesting that it is unlikely that this protein contributes greatly to a protective mechanism against Aβ aggregation and toxicity. The MT-1/2 isoforms however (as exemplified by MT-2A) are broadly expressed within the adult brain, and greatly elevated levels of expression of these proteins have been noted in the AD brain [20]–[22].

We now report that MT-2A is also capable of preventing copper-mediated Aβ aggregation (both Aβ_1–40_ and Aβ_1–42_), and that this involves a specific metal exchange interaction. Hence, Zn_7_MT-2A (but not Cu_10_MT-2A or CaMeMT-2A) prevents soluble Cu(II)-Aβ_1–40_ from forming SDS-insoluble Aβ_1–40_ aggregates. This observation might be explained by the well-characterised ability of higher binding affinity metals (ie: copper) to displace lower affinity metals (ie: zinc) within MT [for review see 33]. Similarly, published reports indicate that Aβ has a Cu(II) binding affinity of between 10^−6^ to 10^−11^ [34], [35], while MT-2A has been reported to have a binding affinity for Cu(II) of 10^−19^ [36]. This supports the proposition that MT-2A is capable of removing Cu(II) ions from Cu(II)-Aβ. Interestingly, we found that apo-MT-2A (metal free MT-2A) was unable to prevent soluble Cu(II)-Aβ from forming SDS-insoluble Aβ aggregates. Apo-MT-2A has a high affinity for free copper initially suggesting that it might be able to extract Cu from Aβ aggregates. It is possible that the apo-MT rapidly becomes oxidised (via disulphide-bond formation between cysteine residues), which would prevent apo-MT from binding to metals. Or it might be that the ability of MT to participate in an inter-molecular interaction with Aβ under biological conditions is dependent on not just the relative copper binding affinity between the two proteins, but also on the tertiary structure of the zinc-metallated form of metallothionein, which is fundamentally different to the uncoordinated structure found in the apo-thionein. Finally, ICP-MS demonstrated that there was almost 40% less copper in the pellet fraction when Cu(II)-Aβ was aggregated in the presence of Zn_7_MT-2A. In summary, we provide strong evidence that a metal exchange between Cu(II)-Aβ and Zn_7_MT-2A is responsible for blocking the aggregation of Aβ into an insoluble form.

In our studies, we noted that Zn_7_MT-2A was effective at 10-fold lower concentrations than Zn_7_MT-3 in preventing copper-mediated Aβ_1–40_ aggregation. We predict that this difference might be related to the relative copper binding affinities of MT-2A and MT-3, and based upon our experiments with chimeric MT proteins we believe that the α-domain of MT-2A is particularly important in this activity. We note that the relative affinity of the α- and β- domains of MT for copper means that the activity of MT-2A and MT-3 towards copper-induced Aβ aggregation will probably depend on the molar ratio of MT to Aβ. In this regard, it is generally considered that the β-domain of MT fills with copper first, followed by the α-domain. We show that MT-2A will prevent aggregation of Cu(II)-Aβ even at low relative levels of this isoform (eg 0.5–5 µM MT to 25 µM Aβ), whereas MT-3 requires a higher MT∶Aβ ratio (25 µM MT to 25 µM Aβ) for activity. This most likely also explains the difference between MT-2A and MT-3 in the neurotoxicity data, in which we used 40µM Cu(II)-Aβ to 20µM MT. We predict that when the ratio of Aβ to MT is equimolar that it will be primarily the β-domains of MT that will be filled with copper, and that in this scenario MT-2A and MT-3 will protect equally against Cu(II)-Aβ neurotoxicity.

We also found that Zn_7_MT-2A protects neurons against Cu(II)-Aβ toxicity. We believe that this involves a zinc/copper metal-exchange between Zn_7_MT-2A and Cu(II)-Aβ that subsequently prevents Aβ-bound copper from participating in redox-reactions and producing reactive oxygen species [as suggested in 23]. Hence, only Zn_7_MT-2A but not Cu_10_MT-2A or CaMeMT-2A was capable of protecting cortical neurons from soluble Cu(II)-Aβ neurotoxicity. Notably, Cu_10_MT-2A itself was not toxic to neurons, indicating that when copper is bound to MT (for instance following metal-swap with Cu(II)-Aβ) that it is unable to produce ROS. As evidence that MT-2A is not acting downstream by scavenging the H_2_O_2_ generated by Cu(II)-Aβ, we found that Zn_7_MT-2A provided only a small degree of protection against direct H_2_O_2_ neurotoxicity. This suggests that Zn_7_MT-2A neuroprotection against Cu(II)-Aβ is primarily afforded through a zinc/copper metal swap and subsequent inhibition of H_2_O_2_ generation. We do note that it is possible that when Cu(II)-Aβ is applied to neurons that some copper is released from the complex, and that this free copper is also partly responsible for neurotoxicity. Although, as noted by Meloni et al [23], we think that only a small amount of copper is released from Cu(II)-Aβ upon addition into the culture medium and the primary cause of neurotoxicity is the Cu(II)-Aβ complex.

Finally, using a direct and sensitive technique, MIFE, to measure the kinetics of ion fluxes, we were able to determine that oxidative stress (H_2_O_2_) generated by soluble Cu(II)-Aβ induces a rapid net efflux of K^+^ and mild net influx of Ca^2+^ into cortical neurons most likely via increased oxidative stress. Notably, K^+^ efflux from cells is a well established trigger of apoptosis. These observations are also in accordance with the results of other groups who have demonstrated that H_2_O_2_ induces substantial dysregulation in neuronal K^+^ channel conductance [37] and calcium homeostasis [38]. Importantly, we found that treatment with Zn_7_MT-2A completely abolished the Cu(II)-Aβ-induced changes in K^+^ and Ca^2+^ net ion fluxes. Taken together, our data suggests that the neuroprotective actions of MT-2A lie in its ability to exchange metals with Cu(II)-Aβ to stop production of ROS, and preventing subsequent detrimental changes in ionic balance within neurons.

We believe that our results may reflect a physiological action of MT-2A in the Alzheimer's brain. For instance, MT-1/2 levels in the adult human brain have been reported to be approximately 40µg/g [39]. Expression is primarily by astrocytes, with very low levels of MT-2A expressed in neurons. We have recently reported that secretion of MT-2A by cultured astrocytes can be induced under certain physiological conditions and that extracellular MT-2A can be detected in the site of a physical injury to the brain [15]. Hence, it is conceivable that under stressful situations MT-2A may be secreted from astrocytes into the synaptic vicinity and reach the levels that we have demonstrated are capable of modulating Cu-mediated Aβ aggregation.

Because of the considerable published data linking metal-binding to the aggregation of Aβ, metal-chelation drugs have been proposed as a potential therapy for AD [40], [41]. An excellent example of this approach is the administration of the copper- and zinc- chelating drug clioquinol, which has been reported to prevent plaque formation in transgenic AD mice [42]. The use of such metal-chelating drugs might not only reduce metal-mediated aggregation of Aβ, but also limit the formation of Cu(II)-Aβ and thus prevent the generation of oxidative stress and subsequent neurotoxicity. One criticism of metal-chelation therapies for AD is that these chelating agents remove metal from Aβ leaving a metal-free Aβ that could feasibly readily bind metals again. Hence, more recently, metal redistribution has been proposed as a more appropriate goal of metal-targeted strategies for AD [41]. In this regard, we believe that MT-2A might represent a possible candidate as a metal-redistribution therapeutic agent, as the metal exchange between MT and Aβ leaves the Aβ in a Zn-bound non-toxic form, and redistributes copper into an inert Cu-MT form.

In summary we provide compelling evidence that MT-2A can protect against copper-induced Aβ aggregation and neurotoxicity. This action of MT-2A appears to involve a metal-swap between Zn_7_MT-2A and Cu(II)-A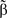 Furthermorė MT-2A can block Cu(II)-Aβ induced changes in ion homeostasis and neurotoxicity of cultured cortical neurons. We propose that there is therapeutic potential in a MT-2A based approach to reducing Aβ deposition in AD.

## Methods

### Metallothionein protein

MT proteins were provided by Bestenbalt LLC (Estonia) as >98% pure HPLC-purified proteins. For this study we have used Zn_7_MT-2A, Zn_7_MT-3, and different metallated forms of MT-2A including Cu_10_MT-2A and carboxymethylated MT-2A (CaMeMT-2A). All MT proteins were provided directly from Bestenbalt in lyophilised form in either a metal free (apo), or in Zn_7_MT or C_10_MT state, Metallation of MT-2A was prepared as we have described previously for Zn_7_MT-3 [43]. Briefly, the protein was dissolved in 20 mM Tris-HCl, pH 8 and the pH was lowered to 2.5. Ten equivalents of Zn^2+^ or 12 equivalents of Cu^+^ was added and the pH raised to 8. The buffer was exchanged to 10 mM ammonium bicarbonate, and the solution frozen at −80°C and freeze dried. The lyophilised proteins were reconstituted in Milli-Q water (pH 7.4) immediately prior to use.

### In vitro copper-mediated Aβ aggregation assay

Synthetic monomeric Aβ_1–40_ and Aβ_1–42_ were purchased from EZBiolab (US). The dried Aβ peptide contains trifluoroacetate (TFA) as a counterion, and in analysis it was found that the synthetic Aβ used in this study contained approximately 5–10% TFA. For *in vitro* Aβ aggregation studies, purified Aβ_1–40_ and Aβ_1–42_ (EZBiolab) was dissolved in Tris buffer (20mM Tris-HCl, 100mM NaCl, pH 7.4) to give a 25µM solution, followed by addition of an equimolar concentration of CuCl_2_ and 200µM ascorbate. The solution was incubated at 37°C for 72hrs with shaking (300 rpm). Aβ aggregation was assessed by gel electrophoresis. Briefly, aggregates were collected by ultracentrifugation at 20,000G for 1 hour then resuspended in LDS (lithium dodecyl sulfate) sample buffer (Invitrogen) and electrophoresed under reducing conditions (β-mercaptoethanol in sample and Invitrogen anti-oxidant supplement in the running buffer) on a 10% Nu-Page Bis-Tris gel (Invitrogen) at 200V for 30 minutes. Protein bands were visualized using Coomassie brilliant blue stain. Aggregates were assessed as SDS-soluble when the Aβ protein was observed in monomeric form on the gel and as SDS-insoluble when not observed on the gel.

### Electrospray ionisation mass spectrometry (ESI-MS) metal binding analysis

Copper binding affinity of MT-3 was determined in strictly similar conditions and by identical approach, which we have recently elaborated for MT-2A [24]. Briefly, 3.3 µM samples of apo-MT-3 were reconstituted with Cu(I) by addition of 12 equivalents of Cu(I)DTT complex in 20 mM ammonium acetate pH 7.5 in the presence of 10 mM DTT. After addition of various concentrations of Cu(I)-chelating reagent diethyl dithio carbamate (DETC), samples were incubated for 2 minutes and injected into the electrospray ion source of QSTAR Elite ESI-Q-TOF MS instrument (Applied Biosystems, Foster City, USA) by a syringe pump at 6 µl/min and ESI-MS spectra were recorded over a 5 minute period in m/Z region from 500–3000 Da at following instrument parameters: ion spray voltage 5500 V; source gas 45 l/min; curtain gas 20 l/min; declustering potential 60V; focusing potential 320 V; detector voltage 2300V.

The Cu-binding affinity of MT-3 was determined from ESI-MS titration results of Cu_12_MT-3 with DETC by correlating the fractional occupancy of Cu(I)-binding sites in MT-3 with the concentration of free Cu(I) ions. The fractional occupancy of Cu(I)-binding sites in MT-3 (Y) was calculated from ESI-MS spectra (considering that there are 12 Cu(I) binding sites in MT-3) by using the following equation:
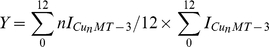
(1)where I_CunMT-3_ denotes the intensity of the Cu_n_MT-3 peak in the ESI-MS spectra. The fractional occupancy of Cu(I) binding sites in MT-3 was correlated with the concentration of free Cu(I) ions in the sample calculated using the apparent dissociation constant for DETC that we have recently determined using the same techniques (K_Cu_ = 13.8 fM) [24]. The obtained binding curve for MT-3, presented in **Fig. 2C**, was fitted nonlinearly with the Hill equation (equation 2) and also linearly to the linear version of the Hill equation with the program “Origin 6.1” (OriginLab Corporation, USA).
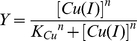
(2)


The nonlinear fitting presented in **Fig. 2C** yielded K_Cu_ values of 0.47±0.01 fM and n values of 1.25±0.06 and n = 1.34 was obtained in linear fitting mode. K_Cu_ is equal to the concentration of free Cu(I) ions at half saturation of MT-3 with Cu(I) ions, reflecting the apparent average affinity of MT-3 towards Cu(I) ions, which is similar to that for MT-2 K_Cu_ values of 0.41±0.02 fM [24]. A Hill coefficient close to 1 indicates that there exists only weak apparent positive cooperativity in the binding of Cu(I) ions to MT-3, whereas strong positive cooperativity (n = 3.3) exists in case of MT-2, which is demetallated in very narrow range of free Cu(I) ions [24]. As seen from **Fig. 2B** there are two metal-thiolate clusters in Cu_12_MT-3, both composed from 6 Cu(I) ions. The first hexacopper-thiolate cluster dissociates readily in the presence of 0.5 mM DETC, whereas DETC can not dissociate copper from Cu_10_MT-2 even at 1 mM concentration [24]. The second hexacopper-thiolate cluster of MT-3 is half desaturated at 3 mM DETC, which is similar to the behaviour of hexacopper-thiolate cluster in MT-2 [24].

### Inductively coupled plasma mass spectrometry (ICP-MS) metal content analysis

In some cases, the Aβ aggregation pellet and supernatant fractions were collected post-ultracentrifugation and their metal content determined by Inductively Coupled Plasma Mass Spectrometry (ICP-MS). Prior to analysis samples were further diluted 10× with ultra-pure water (>18 MOhm) with nitric acid and Indium (as an internal standard) addition to final concentrations 1% and 100 ppb, respectively. Analysis was undertaken using an ELEMENT High Resolution ICP-MS operating in medium resolution mode, enabling ^63^Cu and ^66^Zn isotopes to be monitored free from overlapping spectral interferences. Both elements were quantified using external calibration methodology, with blank subtraction. Typical analytical protocols have been presented previously [44].

### Rodent cortical neural cell cultures

All animal procedures were performed in accordance with the animal ethics guidelines of the University of Tasmania Animal Ethics Committee. Neural cultures were prepared as reported previously [45], and briefly involved the removal of cortices from embryonic day 17 Hooded Wistar embryos, which were incubated with 0.1% trypsin in HEPES buffer at 37°C for 20 minutes. After three washes with warmed HEPES, the tissue was triturated and plated at 5×10^4^ cells per coverslip onto 13mm^2^ glass coverslips in Neurobasal medium (Gibco) and maintained at 37°C in humidified air containing 5% CO_2_.

### Rat cortical neuron toxicity assay

To induce cortical neuron toxicity, 40µM soluble Aβ_1–40_ was applied to neurons in the presence of 40µM CuCl_2_ and 300µM ascorbate. Under these specific conditions, it has been demonstrated that all free copper is rapidly bound by Aβ_1–40_ [23]. The presence of physiological levels of ascorbate permits cycling between copper (II) and (I) oxidation states, as occurs within cellular environments allowing copper to bind to proteins in either Cu(I) or Cu(II) oxidation states. After 24 hours, neuronal viability was measured by the degree of cellular metabolic reduction of alamarBlue®, determined by fluorescence (excitation 535nm, emission 595nm), and was expressed as the percentage of the signal obtained from the vehicle-treated culture. Aβ_1–40_ was used in this study because there is a wider differential in the relative toxicity of copper vs zinc forms of Aβ_1–40_ compared to Aβ_1–42_ (ie: only Cu-Aβ_1–40_ and not Zn-Aβ_1–40_ is neurotoxic, while both Cu- and Zn-Aβ_1–42_ are neurotoxic). Using Aβ_1–40_ thus maximises the effect of the hypothesised metal swap between ZnMT-2A and Cu-Aβ_1–40_, and removes the additional but complicating possibility that metallothionein can independently protect against Zn-Aβ_1–42_ toxicity.

### Statistical analyses of tissue culture experiments

For each experiment unless otherwise stated, a minimum of four wells from at least three separate cultures (derived from different animals), were used for quantification, blinded to conditions. Statistical analysis was completed using SPSS 16.0 (SPSS). When data was unequally distributed, data was transformed so that the residuals were approximately normally distributed. Statistical significance was calculated using One-Way and Two-Way ANOVA with Tukey's Post Hoc Test. All graphical data is presented as mean ± SEM, significance p<0.05.

### Ion-selective flux measurements

The theory of non-invasive microelectrode ion flux (MIFE) measurements was reviewed recently [45] and the complete experimental procedure including ion-selective microelectrode fabrication and cell preparation and immobilisation are given elsewhere [46], [47]. Cortical neurons for the MIFE measurements were grown for three days at a 1×10^5^ cells/well on poly-L-lysine cover slips as described above. By day three a dense monolayer of neurons had developed. Cells were washed in and adapted to the MIFE artificial CSF (aCSF) for one hour prior to experiments. The composition of the aCSF was: 150mM NaCl, 0.5mM KCl, 0.5mM CaCl_2_, 1.5mM MgCl_2_, 1.25mM NaH_2_PO_4_, 5mM NaHCO_3_, 25mM glucose, pH 7.2. Data was acquired at a rate of 15 samples/sec and later averaged over 10 second intervals. Each experiment was repeated upon at least four different coverslips from three different neuronal cultures.

## Supporting Information

Figure S1 Direct cell counting revealed that treatment of 3DIV rat cortical neuron cultures with 40µM Cu(II)Aβ1–40 resulted in significant neuronal death, which could be blocked by the co-addition of 20µM of Zn7MT-2A. Error bars represent standard error of the mean calculated from at least three different experiments. * - p<0.05 (One-Way ANOVA).
(3.21 MB TIF)
