# Supplementary figures and images for: The Native Copper- and Zinc- Binding Protein Metallothionein Blocks Copper-Mediated Aβ Aggregation and Toxicity in Rat Cortical Neurons
Source: PLoS One. 2010 Aug 11;5(8):e12030. doi: 10.1371/journal.pone.0012030 (PMC2920313; doi:10.1371/journal.pone.0012030)

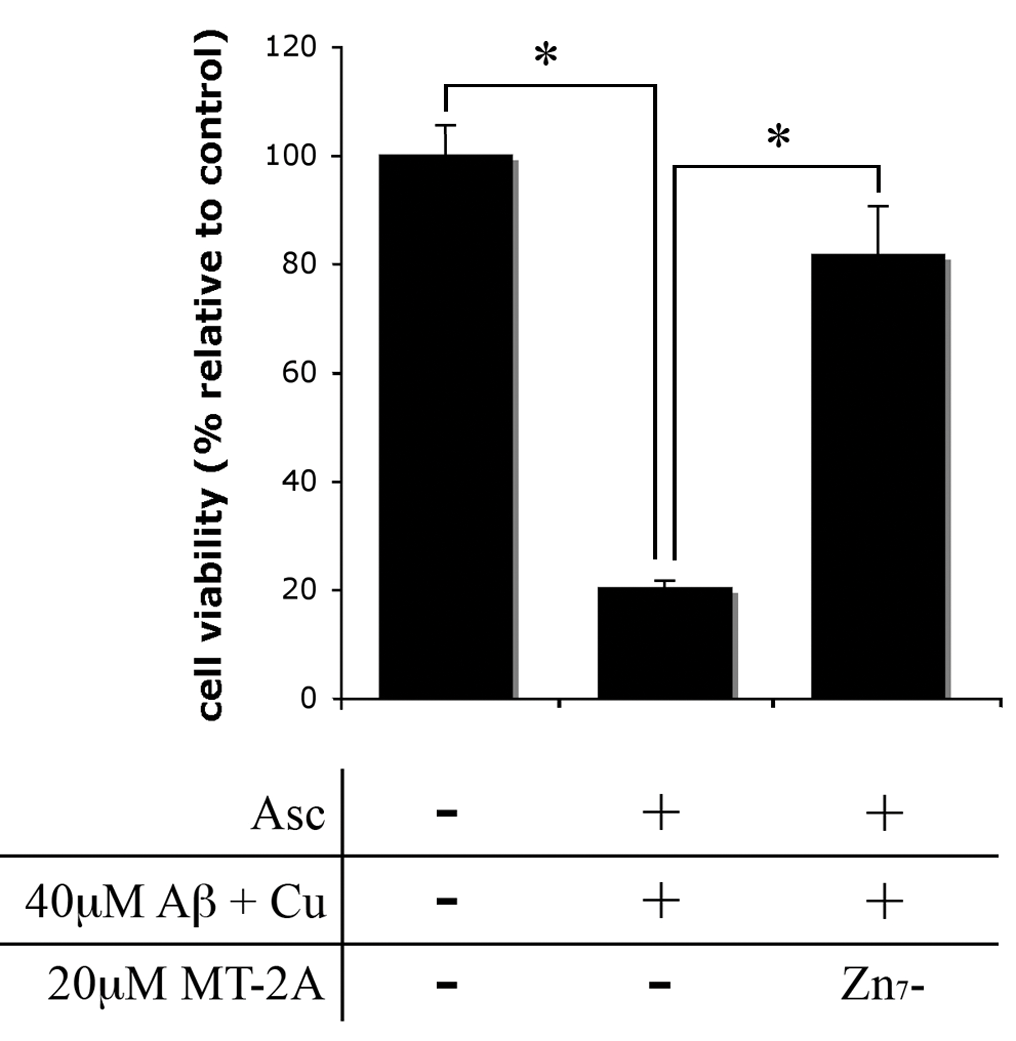

Supplement: Figure S1 — Direct cell counting revealed that treatment of 3DIV rat cortical neuron cultures with 40µM Cu(II)Aβ1–40 resulted in significant neuronal death, which could be blocked by the co-addition of 20µM of Zn7MT-2A. Error bars represent standard error of the mean calculated from at least three different experiments. * - p<0.05 (One-Way ANOVA). (3.21 MB TIF) [file pone.0012030.s001.tif]
